# Comparative Assessment of Human‐ *vs*. Murine‐ derived Microglia as a Model for pro‐Inflammation in Alzheimer’s Disease

**DOI:** 10.1002/alz.085488

**Published:** 2025-01-03

**Authors:** Olivera M. Mitrasinovic

**Affiliations:** ^1^ PCM Consulting, Pathways Connectivity Maps Inc., Mountain View, CA USA

## Abstract

**Background:**

High‐throughput assays have attracted significant attention in Alzheimer’s Disease (AD) research, especially for enabling rapid diagnostics screening for factors at the molecular level contributing to the disease recurrence. With advances in laboratory automation, there is a growing need for quality pre‐clinical data. Assays such as Microarrays, Proteomics, or AI are all dependent on high‐quality input data that serve as a starting point. We have evaluated whether data from murine parallel human microglial cell cultures and have focused our study on three main biomarkers: proinflammatory inducer IL‐1α, neurotoxic iNOS and phagocytosis Fcγ Receptor system implicated in Aβ clearance after antibody mediated opsonization as an immunization paradigm.

**Method:**

Two representative cell lines were included in this study: Human‐derived SV‐A3 microglia, cell count, viability and cytotoxicity matched for comparative analysis to well‐established, SV40 antigen immortalized Mouse BV‐2 as we described (*O. Mitrasinovic et al. J.Biol.Chem. 276 (32): 30142‐9 (2001*)); 24h post‐immune activation expression fold changes were assayed with SYBR/TaqMan qRT‐PCR. Computational AI method validation was done in MATLAB®.

**Result:**

Normalized relative to placebo treated cells by dose‐matched each respective transfection media only, average expression fold increases of IL‐1α in BV‐2 and SV‐A3 had comparable trend, but consistently slightly higher in later: 29.41 *vs*. 36.23. Similar observation was found for neurotoxic iNOS: 2.45 vs. 4.03, demonstrating similar neurotoxic pro‐ inflammatory response. Qualitatively, phagocytosis of Fluorescently labeled FITC‐ Aβ1‐40 was similar. However, differences in the uptake of Antibody‐Opsonized Aβ were prominent, consistently with structural and genetic differences in FcγR’s in between mouse and human microglia.

**Conclusion:**

The extent of induced inflammation in AD demonstrates species‐specificity, and while for a number of genetically conserved factors similarity responses exist, especially main pro‐inflammation and neurotoxicity biomarkers, human cells seem to activate more robust inflammatory responses. Existence of the unique human *vs*. mouse differences at the genetic level for Aβ phagocytic processing factors needs to be taken into consideration in any subsequent AI‐type studies or high‐throughput approximations at the cellular level.

**Acknowledgments**: Alzheimer’s Association for funding, *in part*.

